# Antioxidant Activity of Fucoidan Modified with Gallic Acid Using the Redox Method

**DOI:** 10.3390/md20080490

**Published:** 2022-07-29

**Authors:** Keylla Dayanne Coelho Marinho de Melo, Lucas dos Santos Lisboa, Moacir Fernandes Queiroz, Weslley Souza Paiva, Ana Carolina Luchiari, Rafael Barros Gomes Camara, Leandro Silva Costa, Hugo Alexandre Oliveira Rocha

**Affiliations:** 1 Postgraduate Programe Health Sciences, Federal University of Rio Grande do Norte (UFRN), Natal 59.078-970, RN, Brazil; keylladayanne@hotmail.com; 2 Department of Biochemistry, Federal University of Rio Grande do Norte, Natal 59.078-970, RN, Brazil; lslisboa.1@gmail.com (L.d.S.L.); moacirfqn@gmail.com (M.F.Q.); wdspaiva@gmail.com (W.S.P.); rafael_bgc@yahoo.com.br (R.B.G.C.); 3 Laboratory of Ornamental Fish, Department of Physiology and Behavior, Bioscience Center, Federal University of Rio Grande do Norte, Natal 59.078-970, RN, Brazil; luchiarilab@gmail.com; 4 Federal Institute of Education, Science and Technology of Rio Grande do Norte, Canguaretama 59.190-000, RN, Brazil; leandro-silva-costa@hotmail.com

**Keywords:** sulfated polysaccharides, oxidative damage, sulfated fucan, antioxidant activity

## Abstract

Antioxidant compounds decrease the amount of intracellular reactive oxygen species (ROS) and, consequently, reduce the deleterious effects of ROS in osteoblasts. Here, we modified a 21 kDa fucoidan (FucA) with gallic acid (GA) using the redox method, to potentiate its antioxidant/protective capacity on pre-osteoblast-like cells (MC3T3) against oxidative stress. The 20 kDa FucA-GA contains 37 ± 3.0 mg GA per gram of FucA. FucA-GA was the most efficient antioxidant agent in terms of total antioxidant capacity (2.5 times), reducing power (five times), copper chelation (three times), and superoxide radical scavenging (2 times). Exposure of MC3T3 cells to H_2_O_2_ increased ROS levels and activated caspase-3 along with caspase-9. In addition, the cell viability decreased approximately 80%. FucA-GA also provided the most effective protection against oxidative damage caused by H_2_O_2_. Treatment with FucA-GA (1.0 mg/mL) increased cell viability (~80%) and decreased intracellular ROS (100%) and caspase activation (~80%). In addition, Fuc-GA (0.1 mg/mL) abolished H_2_O_2_-induced oxidative stress in zebra fish embryos. Overall, FucA-GA protected MC3T3 cells from oxidative stress and could represent a possible adjuvant for the treatment of bone fragility by counteracting oxidative phenomena.

## 1. Introduction

Structurally, fucoidans have a large amount of sulfated α-L-fucose, which form a linear backbone, and other monosaccharides (mannose, galactose, glucose, uronic acid, and xylose) in smaller amounts, which form branches [1]. The bond between the monosaccharide units can be (1→3), but alternating bonds between (1→3) and (1→4) can be found, all of which can be alpha or beta, depending on the type of monosaccharide [2,3]. Although the sulfation of fucoidan is variable, as is the degree of sulfation, the sulfate groups are mainly attached to the C2 and C4 carbons and occasionally to the C3 carbon of fucose residues [4].

It is worth noting that this description of fucoidan mainly represents data obtained from the fucoidans synthesized mainly by seaweeds from the order Fucales (e.g., *Fucus vesiculosus* and *Ascophyllum nodosum*) and Laminariales (e.g., *Saccharina japonica*, *Undaria pinnatifida*, and *Laminaria saccharina*). This information does not truly represent the structural complexity of fucoidans, especially those found in other orders of brown seaweeds. Other fucoidans have emerged with much more complex and different structures than those that fit the above description [5]. For example, the fucoidan from the seaweed *Spatoglossum schröederi* is known as FucA. It has a central structure composed of glucuronic acid; fucose is present in the branches, and some xyloses are sulfated [6].

Fucoidans have been identified as agents with diverse biological and pharmacological properties [7], and several studies have indicated the importance of the sulfate groups present in these fucoidans for their anticoagulant [8], antiviral [9], antitumor [10], anti-inflammatory [11], immunostimulant, and antioxidant [12] activities.

Brown seaweeds synthesize other antioxidant compounds, e.g., phlorotannins, such as dieckol, eckol, eckstolonol, phloroglucinol, phlorofucofuroeckol, and 8′,8′-bieckol, 2,7″-phloroglucinol-6,6′-bieckol [13], and carotenoids such as fucosterol, α-tocopherol, and fucoxanthin [14]. However, these compounds are synthesized in low quantity and their purification process is expensive and generates chemical residues that are harmful to the environment [15,16]. On the other hand, fucoidans are synthesized in large quantities by seaweeds, they are water soluble, they are non-toxic, and the fucoidan purification methods are more environmentally friendly [17,18,19]. 

The antioxidant activity attributed to fucoidans was first described by Athukorala et al. [19]. Since then, several studies on antioxidant fucoidans have been published, and several recent papers have reviewed this subject, such as those by Kalita et al. [20], Wang et al. [21], Yang et al. [22], and Pradhan et al. [12]. The mechanism of action of fucoidans is based on neutralizing cellular damage or the intermediary pathways involved in the formation of reactive species [21].

Antioxidant is a term used to define any compound that can pro-oxidant agents that damage DNA, cell membranes, and other parts of cells. Oxidative stress occurs when there is an increase in the number of reactive species and/or a decrease in the activity of antioxidant agents. This is caused by pro-oxidant agents including free radicals, ions, or unstable molecules, which contain an unpaired electron in their valence shell. Under normal physiological conditions, oxidative stress is countered by endogenous antioxidants [17,18]. However, when antioxidant defenses are overcome, these agents interact with molecules in the body and cause cellular damage, leading to the emergence or progression of various diseases such as neurodegenerative diseases [23], type 2 diabetes, atherosclerosis [24], premature aging, cancer [17], and osteoporosis [25].

Reactive oxygen species (ROS) induce osteoblast differentiation into osteoclasts that lead to an imbalance in this homeostasis (ratio osteoblasts/osteoclast), and therefore diseases that results in the reduction of bone mineral density, such as osteoporosis, may arise [26]. In other words, oxidative stress and osteoblast cell have an important role in this disease [27].

Because ROS are mainly responsible for the deregulation of osteoblastic activity, the natural antioxidant role in the differentiation of osteoblasts have been studied [28]. Nevertheless, because none of the commercial antioxidants show ideal antioxidant properties, there is always a need to find new antioxidants that can adapt to new situations and/or replace existing ones [29]. To this end, fucoidans from brown seaweeds have been actively studied in this context. These polysaccharides can stimulate mesenchymal cell differentiation into osteoblasts without toxic effects [30,31] and have antioxidant activity [5,12].

**Figure 1:**
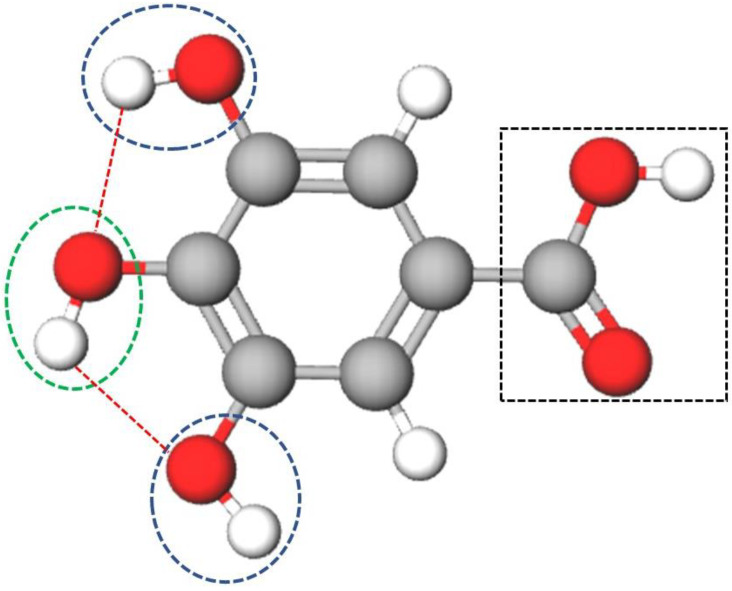
Structure of gallic acid proposed by Dai and Mumper, 2010 [39]. Atoms are represented as spheres with conventional color coding: hydrogen (white), carbon (grey), oxygen (red). The carboxyl group is represented inside the dashed black rectangle. The hydroxyl groups are shown inside the dashed circles; *para* hydroxyl (green dashed circle), *meta* hydroxyl group (blue dashed circle). The hydrogen bonds between adjacent hydroxyl groups are showed as dashed red lines.

However, not all fucoidans are effective antioxidants. For example, FucA has low antioxidant activity [32]. However, this fucoidan is neither genotoxic nor mutagenic [7], and it does not exhibit toxicity in vivo [33]. Therefore, efforts can be made to improve the antioxidant activity of this fucoidan by conjugating with another antioxidant molecules such as gallic acid (Figure 1).

Polyphenols are gaining increasing interest because of their potential health benefits, such as their antioxidant, anticancer, antibacterial, and bone-stimulating capabilities. Phenolic acids are considered powerful antioxidants in vitro and have proven to be more potent than vitamin C, vitamin E, and carotenoids [34]. Phenolic acids can be divided into two classes: benzoic acid derivatives such as gallic acid (GA) and cinnamic acid derivatives, which are synthesized from phenylalanine in living organisms [6]. Gallic acid (3,4,5-trihydroxy benzoic acid) is synthesized by several plants and has antioxidant activity as well as the ability to inhibit lipid peroxidation [35]. It has hydroxyl and carboxyl groups that allow its conjugation to other molecules, enhancing its antioxidant activities [36]. The presence of three hydroxyl groups (two *meta* substituted and one *para* substituted –OH groups) close to the benzene ring increases the solubility of the molecule conjugated to GA (Figure 1) along with the reducing power of the compound [37]. However, polyphenols have low bioavailability and stability, which causes difficulties in their clinical application [38,39]. Therefore, conjugation of gallic acid with sulfated polysaccharides to increase its bioavailability and potentiate the antioxidant activity of the compound needs to be explored.

Gallic acid can be conjugated to polysaccharides using several methods [40]. Of these, the redox method is advantageous as it does not generate toxic reaction products and can be carried out at room temperature to avoid the degradation of antioxidants [41]. Therefore, in the present study, FucA obtained from the seaweed *S. schröederi* was modified with GA using the redox method, and its antioxidant activity was evaluated in the oxidative process. In addition, FucA and its GA-conjugate derivate were evaluated regarding whether they could protect pre-osteoblastic (MC3T3) cells from oxidative damage.

## 2. Results and Discussion

### 2.1. Physicochemical Characterization of Fucoidans

FucA was conjugated to gallic acid, and the product of this process was named FucA-GA. The physicochemical characteristics of these compounds were analyzed, and the data are shown in Table 1.

Table 1 shows that the monosaccharide composition of the two polysaccharides is very similar. However, there is a significant difference (approximately 2.4%) between the sugar content of FucA and that of FucA-GA, which was reflected in the molecular weight of FucA-GA. This is probably because the loss of monosaccharides in FucA-GA was compensated for by the addition of GA molecules. The conjugation method used in our study is not very aggressive; however, it causes the loss of molecular weight of polysaccharides. Wu et al. [42] and Queiroz et al. [43], who conjugated chitosan with GA, reported a loss of 10% and 25%, respectively, which was much higher than that observed with FucA. 

It is not yet clear as to why FucA was resistant to this loss. Chitosan is a linear polysaccharide and FucA is a branched polymer. This could be the explanation for the smaller break observed in FucA. This possibility is corroborated by the fact that laminarin, a branched polysaccharide, when conjugated with gallic acid, also showed little break, less than 2%, in its structure [44]. In addition, FucA is a branched sulfated polysaccharide, and its sulfate groups are found in fucose and xyloses residues, which, in turn, are found in the branches of FucA. Therefore, we believe that these structural features, branches made up of sulfated monosaccharides, have protected FucA from breaks caused during the conjugation process. In the future, we aim to remove the sulfate groups of FucA and verify if there is a greater breakdown of the polysaccharide when subjected to conjugation with gallic acid.

Further, phenolic compounds were observed in FucA-GA. These compounds were not found in FucA, indicating that they are derived from GA, which reveals that GA was covalently bound to FucA after the conjugation process.

Paiva et al. [45] used the redox method to conjugate chitosan with gallic acid and verified the presence of 4% phenolic compounds in the conjugated chitosan, a value similar to that found in FucA-GA. However, chitosan is a polysaccharide resulting from the deacetylation of chitin and has two possible binding sites for GA; FucA has six more sites due to the presence of xyloses and fucoses in the side chains. Despite this, the amount of GA in the FucA-GA molecule was not higher than that found in chitosan.

It is not yet clear which factors affect the amount of GA that is conjugated to a polysaccharide. Studies with chitosans have suggested that the size of the molecule is an important factor. Larger molecules have more GA binding sites than smaller molecules; consequently, more GA is conjugated to the molecule [41,46]. This also seems to be valid for dextrans, as Queiroz et al. [47] and Vittorio et al. [48] reported that three times more GA conjugated to a 15 kDa dextran than to a 4 kDa dextran.

However, chitosans and dextrans are linear polysaccharides, whereas FucA is a branched polysaccharide. This characteristic of FucA could have a greater influence on the amount of GA conjugated to FucA than on its molecular mass. This possibility is corroborated by the fact that when a laminarin (a beta glucan from brown seaweed) was conjugated with gallic acid, using the same process described here, it presented only 1.2% of gallic acid conjugated to its structure [44]. In the future, we intend to identify the characteristics of FucA that possibly influence the conjugation to GA, to optimize this process.

### 2.2. Analysis of In Vitro Antioxidant Activity

Antioxidants are substances that delay, prevent, or remove oxidative damage to a target molecule, or neutralize or prevent the oxidation of substrates [49]. Preventive antioxidants prevent the formation of reactive species by inhibiting pro-oxidant enzymes or participating in the chelation of metal ions. Chain blockers directly eliminate reactive species, and repairers serve to correct oxidative damage [50]. Therefore, the antioxidant activities of FucA and FucA-GA were evaluated using different in vitro methods to identify the type of antioxidant that defines FucA-GA.

#### 2.2.1. Copper and Ferrous Ions Chelating Ability

FucA and FucA-GA samples were evaluated for their ability to chelate ferrous ions at different concentrations (0.1 to 2.0 mg/mL). However, neither FucA nor FucA-GA showed any iron-chelating activity under the set conditions (data not shown). Laminarin [44] as well as dextran [47], when conjugated to GA, did not show an increase in iron-chelating activity either. Notably, GA has been shown to exhibit low iron-chelating activity [51].

**Figure 2:**
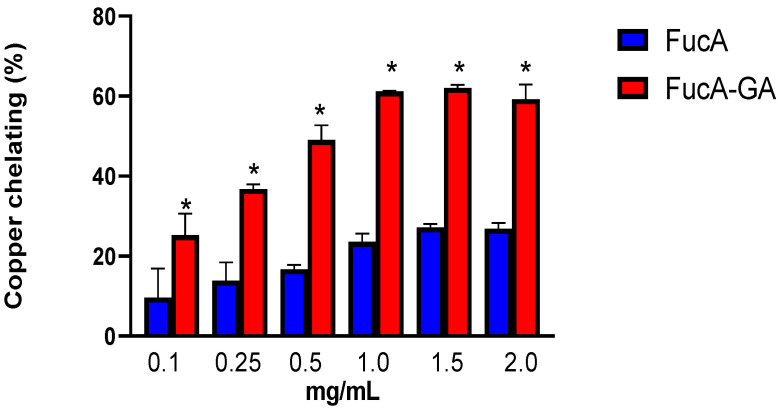
Copper chelating activity of polysaccharides. * indicates significant difference between the FucA and FucA-GA samples at the same concentration (*p* < 0.05). Statistical analysis was performed using one-way ANOVA.

In contrast, both molecules were able to chelate copper (Figure 2). However, their activities also differed. FucA (1.0 mg/mL) showed a maximum activity of approximately 30%, which did not increase, even when FucA activity was evaluated at twice the concentration (2.0 mg/mL). FucA-GA had higher activity than FucA under all the conditions evaluated. Furthermore, similar to FucA, FucA-GA reached its maximum activity at a concentration of 1 mg/mL; however, in this case, the activity of FucA-GA was twice that observed with FucA.

The GA activity in this test was also evaluated. In this case, 0.037 and 0.074 mg/mL were chosen, which would correspond to the amounts of gallic acid present in FucA-GA 1.0 mg/mL and 2.0 mg/mL, respectively. In both cases, the GA activity reached 100%.

It has already been shown that GA has copper-chelating activity [52]. Therefore, it is believed that part of the GA chelating activity was transferred to FucA-GA, which explains the higher copper-chelating activity. Furthermore, the copper chelating activity of FucA-GA was superior to that of other GA-conjugated polysaccharides such as GA-conjugated chitosan (0.5 mg/mL), which exhibited approximately 20% copper chelation [45]. A possible explanation for this is the conformation that FucA-GA assumes after conjugation. This would facilitate greater interaction with copper ions and, at the same time, make it difficult for FucA-GA to interact with iron ions. In addition, FucA-GA was a more potent copper-chelating agent than fucoidan (2.5 mg/mL) from brown seaweed *Undaria pinnatifida* [53], sulfated glucan (from 0.1 to 1.0 mg/mL) from brown seaweed *Dictyopteris justii* [54], and fucoidans (from 0.1 to 2.0 mg/mL) from *Dictyota mertensii* [55].

The accumulation of copper in the body, due to excessive consumption, intoxication, or genetic alterations, such as in Wilson’s disease and Menkes syndrome, leads to oxidative stress, which culminates in apoptosis of cells in different organs, such as the brain, kidneys, and corneas [56,57,58]. To combat this problem, reduction of copper intake and administration of chelating agents (e.g., D-penicillamine or trientine) that increase urinary copper excretion are promoted [59]. However, there is always a need for new chelating agents that can replace existing ones. The data obtained here confirm that FucA-GA has the potential to be evaluated in vivo as a copper-chelating agent.

These data also showed that FucA-GA can be classified as a preventive antioxidant, as it is a copper-chelating agent.

#### 2.2.2. Evaluation of Hydroxyl (OH) Radical Scavenging

**Figure 3:**
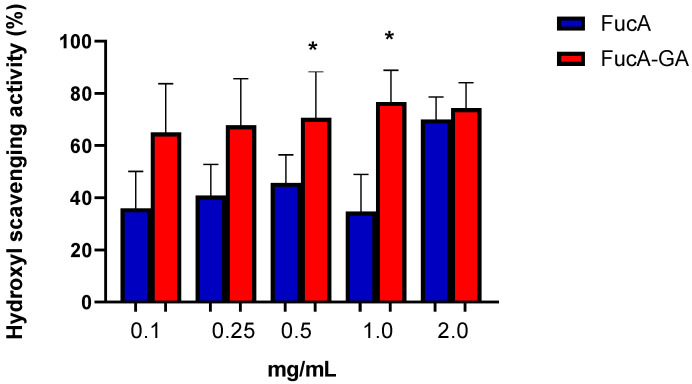
Hydroxyl scavenging activity. * indicates significant difference between the FucA and FucA-GA samples (*p* < 0.05).

Both fucoidans showed hydroxyl radical scavenging activity (Figure 3). However, FucA-GA had the highest activity (~80%) at the lowest concentration. This value did not change with increasing concentrations of FucA-GA. Maximum FucA activity was reached at a concentration of 2.0 mg/mL, and at this concentration the hydroxyl radical-scavenging activities of the two fucoidans were statistically similar.

The values obtained with FucA-GA and FucA (1.0 and 2.0 mg/mL) are close to that described (75.2%) for the non-commercial *U. pinnatifida* fucoidan [5], indicating that the fucoidan from this seaweed is a good hydroxyl ion-scavenging agent. However, Yu et al. [60] reported commercial *U. pinnatifida* fucoidan with 30% hydroxyl radical scavenging activity; this seems to indicate that the product batch can also influence the observed antioxidant activity. In addition, these variations in fucoidans batch are common and are also related to factors such as seasonality, collection location, seaweed age, and fucoidan extraction/purification method [8,9,10,11,12].

Several authors have reported the hydroxyl radical-scavenging activity of GA [37,40,61]. However, the conjugation of GA with a polysaccharide is not always able to transfer this activity to the formed compound. For example, the laminarin from the seaweed *Lobophora variegata* was conjugated to GA but did not show hydroxyl radical-scavenging activity, even at high concentrations (2.0 mg/mL). Hou et al. [62] reported that the molecular mass and spatial structure of fucoidans are decisive factors for their hydroxyl radical scavenging ability. Therefore, it is believed that the conformation that FucA-GA assumed after conjugation allowed the hydroxyl radical scavenging activity to occur. Additionally, these data indicated that FucA-GA is also a chain blocker that acts as an antioxidant compound to directly eliminate reactive species (hydroxyl radical).

#### 2.2.3. Assessment of Reducing Power and Determination of Total Antioxidant Capacity (TAC)

**Figure 4:**
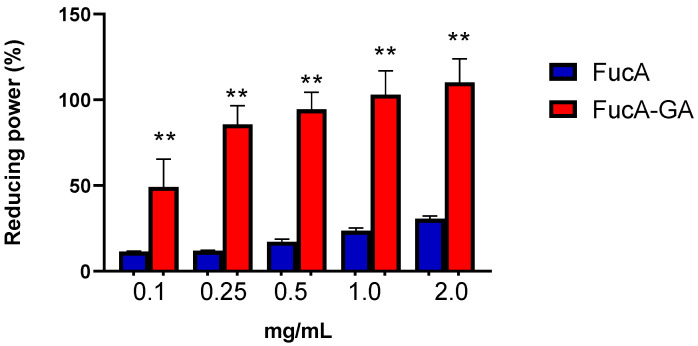
Reducing power. ** indicates a significant difference between the FucA and FucA-GA (*p* < 0.0001).

The reducing power assay depends on the reduction of potassium ferricyanide by the samples and demonstrates the ability to give up electrons at a pH close to neutral [34]. Figure 4 shows that the reducing power of FucA-GA was greater than that of FucA under all the conditions evaluated. The activity of FucA does not exceed 25%, even at the highest concentration (2.0 mg/mL). In contrast, FucA-GA, reached 100% activity at 0.5 mg/mL. On the other hand, GA (0.037 and 0.074 mg/mL) showed no activity in this test.

**Figure 5:**
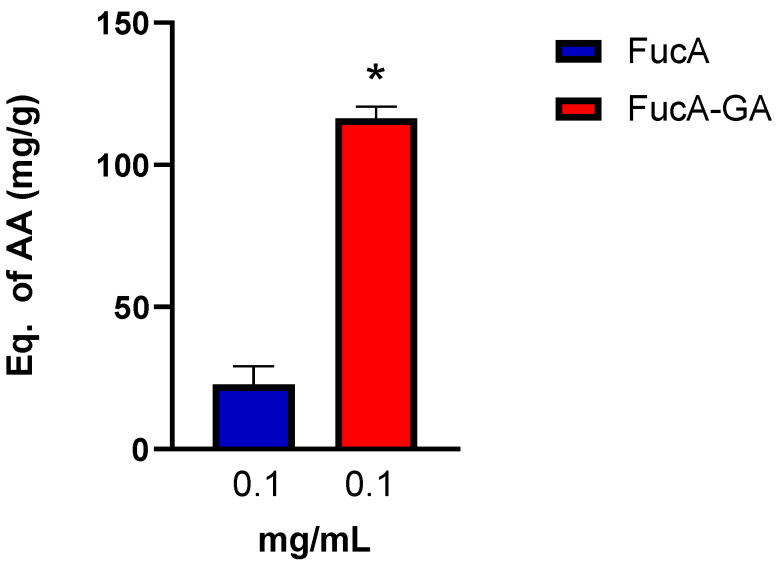
Total antioxidant capacity (TAC) assay of FucA and FucA-GA. * indicates significant difference between the FucA and FucA-GA samples (*p* < 0.0001).

The ability of FucA and FucA-GA to donate electrons was also evaluated using the total antioxidant capacity (TAC) assay, because this test evaluated the donation of electrons in an acidic medium [34]. FucA-GA showed superior activity to FucA in the TAC assay. In addition, this FucA-GA activity was higher than those found in commercial fucoidan from *U. pinnatifida* [5] and fucoidan from *Fucus vesiculosus* [63]. These results indicate that FucA-GA can be a good electron donor under different pH conditions (Figure 5). In this test, GA (0.037 and 0.074 mg/mL) did not show antioxidant activity.

Gallic acid contains three OH groups: one *meta*-, and two *para*-substituted OH groups (Figure 1). These hydroxyl groups affect antioxidant ability by intramolecular hydrogen bonding; in addition, they tend to stabilize the antioxidant radical formed [34]. It is suggested that the *para*-substituted –OH groups form hydrogen bonds with the *meta*-substituted –OH group, resulting in a lower hydrogen bond dissociation enthalpy and hence can stabilize this group after it has donated one of its electrons. This feature makes GA a good antioxidant [64]. In addition, the electron donation efficiency of GA depends on its steric freedom, which in turn depends on its substituent [35].

Curcio et al. [65] suggested that GA binds to polysaccharides through its carboxyl group, forming an ester with the polysaccharide at the end of the process. When chitosans [65] and dextrans [47] were conjugated with gallic acid, they showed good antioxidant activity in the reducing power test but low activity in the TAC assay. These two tests were performed at different pH values, which can change the polysaccharide conformation and, consequently, the steric freedom of the GA. This could explain the difference in the activity of these polysaccharides in these two different tests, despite the same property being evaluated, i.e., the ability to donate electrons.

Chitosans and dextrans are linear polysaccharides, whereas FucA-GA is not a linear polysaccharide. Furthermore, FucA-GA has sulfate groups. These characteristics may have allowed FucA-GA to provide a microenvironment for GA to exert its antioxidant activity completely, including giving the GA steric freedom and, therefore, giving the GA-conjugated FucA the ability to be a good electron donor. However, identifying these characteristics was beyond the scope of this paper. Future studies are warranted to evaluate these characteristics and to determine the correlation between the structure and the antioxidant activity of FucA-GA.

To assess the stability of FucA-GA, a solution of this polysaccharide was kept at 4 °C for 12 months. After this period, FucA-GA (1.0 mg/mL) was evaluated as an antioxidant agent by means of three in vitro tests (copper chelating activity, reducing power assay, and TAC). In all, no significant difference was found in relation to the values shown in Figure 3, Figure 4 and Figure 5, respectively. This indicates that FucA-GA remains stable for at least 12 months.

### 2.3. Effect of FucA and Fuc-GA on Pre-Osteoblastic Cells MC3T3

Oxidative stress regulates cell functions under unfavorable conditions, including impairment of bone formation. Reactive Oxygen Species (ROS) are identified as one of the main causes of bone cell hemostasis breakdown as ROS lead to apoptosis of osteoblasts or differentiation of osteoblasts into osteoclasts, which promotes bone resorption and the onset of diseases such as osteoporosis [66].

Fucoidans from *Undaria pinnatifida*, *Sargassum muticum*, and *Turbinaria ornata* can promote osteogenesis by reducing the presence of intracellular ROS, thus reducing the deleterious effects of these reactive species on osteoblasts [67]. Therefore, we investigated whether FucA and FucA-GA could protect pre-osteoblastic cells from oxidative damage.

The effects of FucA and FucA-GA on the viability and proliferation of MC3T3 cells (osteoblast precursor cell line derived from *Mus musculus*) were evaluated. Subsequently, the possibility of these compounds protecting the cells against oxidative stress caused by hydrogen peroxide (H_2_O_2_) was also evaluated.

#### 2.3.1. Cytotoxicity Assessment and Cell Death Induction by FucA and FucA-GA on MC3T3 Cells

The first experiment was performed to assess whether the polysaccharides were cytotoxic to the MC3T3 cells. For this, the MTT [3-(4,5-Dimethylthiazol-2-yl)-2,5-diphenyltetrazolium bromide] assay was used. This compound is metabolized by the mitochondrial/cytoplasmic enzymes in cells [68].

None of the polysaccharides evaluated (0.001 to 2.0 mg/mL) decreased the ability of cells to reduce MTT (data not shown). These data indicated that FucA and FucA-GA did not induce cytotoxicity in MC3T3 cells under the test conditions. The cytotoxicity of GA against MC3T3 was also evaluated. No significant difference (*p* > 0.0001) in cell ability to reduce MTT (%) compared to control cells was found when GA, in the range 0.01 to 0.1 mg/mL, was added.

Subsequently, the proliferation of MC3T3 cells in the presence of FucA and FucA-GA was evaluated by the BrdU (5-bromo-2′-deoxyuridine) incorporation test. This method quantifies cell proliferation based on measuring BrdU incorporation during DNA synthesis in replicating cells [69].

**Figure 6:**
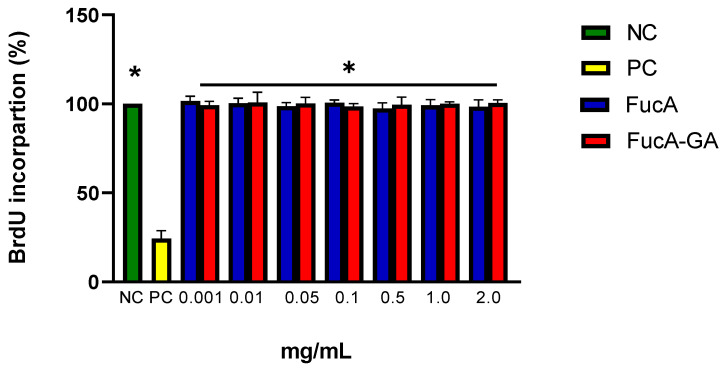
Effect of different concentrations of FucA and FucA-GA on the ability of MC3T3 cells to incorporate BrdU. NC—negative control composed only of culture medium with fetal bovine serum. PC—positive control composed of culture medium with fetal bovine serum and cisplatin (2 µg/mL). * indicates that there is a difference between the polysaccharide samples and PC (*p* < 0.0001).

The presence of polysaccharides, regardless of the concentration, did not affect the ability of the cells to incorporate BrdU (Figure 6). In summary, the data indicated that polysaccharides were neither cytotoxic nor did they affect the proliferation of MC3T3 cells.

Regarding GA, there was also no alteration in the incorporation of BrdU by MC3T3 cells when they were exposed to GA (from 0.01 to 0.1 mg/mL). These data corroborate those described by Sakagami and Satoh et al. [70]. These authors showed that GA is not cytotoxic against human lymphocytes. Some authors have shown that GA (from 0.01 to 0.07 mg/mL) [71] (from 0.051 to 0.136 mg/mL) [72] is cytotoxic and induces cell death in tumor cells. However, in this study, it was observed that GA is neither cytotoxic nor induces cell death of MC3T3 cells. This indicate that the GA cytotoxic effect depends on the GA concentration and may be cell specific.

#### 2.3.2. Induced Oxidative Stress Assay with MC3T3 Cells

**Figure 7:**
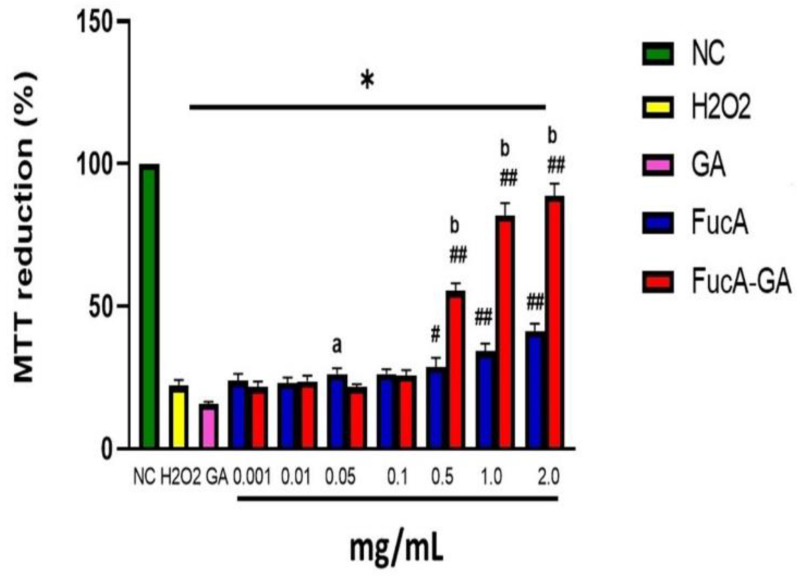
Effect of different amounts (mg/mL) of FucA and FucA-GA on the ability of MC3T3 cells to reduce MTT in the presence of H_2_O_2_. NC—negative control composed only of culture medium with fetal bovine serum. H_2_O_2_—positive control composed of culture medium with fetal bovine serum and H_2_O_2_ (500 µM, final concentration). GA—gallic acid (0.074 mg/mL). * *p* < 0.0001 vs. NC. # *p* < 0.001 vs. H_2_O_2_. ## *p* < 0.0001 vs. H_2_O_2_. (a) indicates a significant difference between FucA and FucA-GA samples (*p* < 0.05) at the same concentration. (b) indicates a significant difference between FucA and FucA-GA samples (*p* < 0.0001) at the same concentration.

The question was, if the polysaccharides do not affect MC3T3 cells, could they also protect them from oxidative damage, as they have antioxidant activity. To answer this question, MC3T3 cells were exposed to H_2_O_2_ in the presence and absence of polysaccharides. The data obtained are shown in Figure 7. Cells not exposed to peroxide (NC) were able to reduce MTT (~100%). In contrast, the ability of cells exposed to peroxide (500 µM, final concentration) to act on MTT was reduced to approximately 20%.

Both FucA (0.001 to 0.1 mg/mL) and FucA-GA (0.001 to 0.1 mg/mL) were not able to protect the cells from peroxide damage. However, FucA-GA at the highest concentrations evaluated (from 0.5 two 2.0 mg/mL) protected cells from oxidative damage. Additionally, in the presence of FucA-GA (1.0 and 2.0 mg/mL), the MTT reduction reached ~80%, which was very close to that observed in the NC group. This data shows that, as seen in the in vitro tests, FucA-GA had greater antioxidant activity than FucA. In addition, FucA-GA showed a stronger effect than the other fucoidans obtained from *F. vesiculosus* and *D. mertensii* [55].

Several papers show GA acting as an antioxidant and protecting cells from H_2_O_2_-induced oxidative stress [73,74,75]. On the other hand, some authors show the opposite effect. For example, Kan et al. [76] demonstrated that GA (from 42.5 to 85 mg/mL) was unable to protect rat neuronal cells (PC12) from oxidative stress caused by the presence of H_2_O_2_. Here, GA failed to protect MC3T3 cells from the damage caused by hydrogen peroxide, which corroborates Kan’s data [76] and shows that the protective effect of gallic acid depends on the cell type. Discussing why GA has this dual effect is beyond the scope of this work. Therefore, because GA did not protect MC3T3 cells from damage caused by peroxide, it was not used in the following experiments.

The fact that FucA-GA protected cells from oxidative damage, while GA failed, shows that the conjugation of fucoidan with GA potentiated the antioxidant action of FucA. In addition, it modifies the action of the GA. These data show that one of the objectives of this work, to obtain a conjugate with better properties than the original compounds (Fuca and GA) was achieved.

#### 2.3.3. Intracellular Reactive Oxygen Species Production

As fucA-GA 1.0 and 2.0 mg/mL showed similar effects (Figure 7), only FucA-GA (1.0 mg/mL) was used in the following tests.

**Figure 8:**
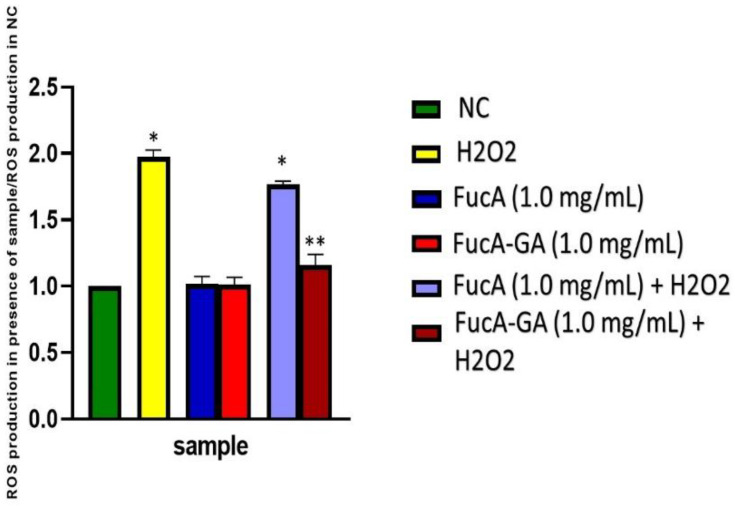
Production of reactive oxygen species (ROS) by MC3T3 cells exposed to H_2_O_2_ (500 µM, final concentration) oxidative stress. The data presented correspond to the mean ± standard deviations (*n* = 3). NC—negative control composed only of culture medium with fetal bovine serum. * *p* < 0.0001 vs. NC. ** *p* < 0.001 vs. NC.

To evaluate the antioxidant effect of FucA and FucA-GA on MC3T3 cells, their effect on the amount of intracellular ROS produced in cells exposed to H_2_O_2_ was evaluated. The results are shown in Figure 8. The exposure of cells to 500 μM H_2_O_2_ greatly increased the ROS production. It was also observed that the intracellular ROS production was not affected in cells only exposed to polysaccharides. In the presence of FucA (1.0 mg/mL), however, there was a decrease in ROS (~20%) compared with that in cells exposed to peroxide. However, FucA-GA (1.0 mg/mL) was much more effective in reducing the amount of ROS in cells exposed to peroxide. There was no significant difference between the ROS concentrations found in the cells exposed to Fuc-GA and those found in the cells of the control group.

#### 2.3.4. Effect of FucA and FucA-GA on Caspase-3 and Caspase-9 in MC3T3 Cells

In addition, some studies have shown that H_2_O_2_ induces cell death through the activation of caspases. Therefore, the activities of caspase-3 and caspase-9 in MC3T3 cells exposed to H_2_O_2_ and polysaccharides (1.0 mg/mL) were evaluated.

**Figure 9:**
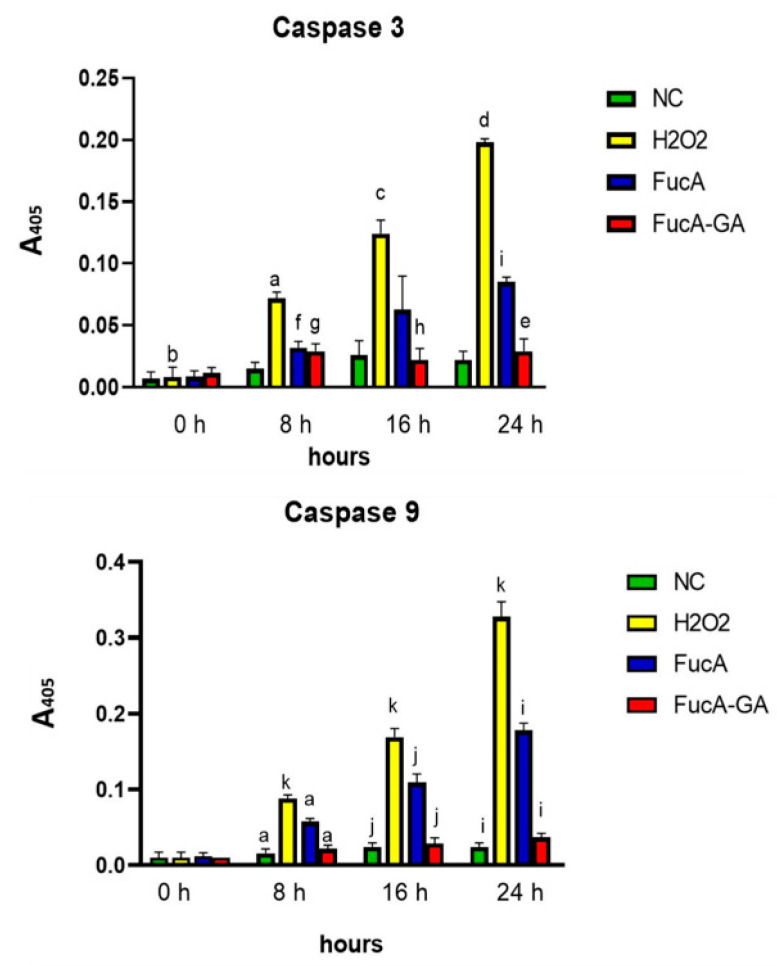
Effect of FucA (1.0 mg/mL) and FucA-GA (1.0 mg/mL) on caspase-3 and caspase-9 activity in MC3T3 cells exposed to H_2_O_2_-induced oxidative stress. H_2_O_2_ was used at 500 µM (final concentration). NC—negative control composed only of culture medium with fetal bovine serum. (a) *p* < 0.0001 vs. NC 8 h. (b) *p* < 0.05 between the samples of H_2_O_2_ 0 h and H_2_O_2_ 8 h; 16 h and 24 h. (c) *p* < 0.01 vs. NC 16 h. (d) *p* < 0.01 vs. NC 24 h. (e) *p* < 0.05 vs. H_2_O_2_ 24 h. (f) *p* < 0.0001 vs. H_2_O_2_ 8 h. (g) *p* < 0.01 vs. H_2_O_2_ 8 h. (h) *p* < 0.01 vs. H_2_O_2_ 16 h. (i) *p* < 0.001 vs. H_2_O_2_ 24 h. (j) *p* < 0.001 vs. H_2_O_2_ 16 h. (k) *p* < 0.001 vs. H_2_O_2_ 0 h. The caspase activity was monitored at the times indicated by measuring the absorbance at 405 nm (A405).

Figure 9 shows that the activity of these caspases increases over time when cells are exposed to oxidative stress (H_2_O_2_-500 µM). In contrast, the presence of polysaccharides decreases the activation of caspases. However, FucA was less effective than FucA-GA. There was no significant difference between the data obtained with the cells exposed to FucA-GA and those obtained with the cells of the control group (NC).

Oxidative damage caused by H_2_O_2_ increased the activation of caspases and decreased the viability of cells, whereas FucA-GA reverses this effect. These data demonstrated that FucA-GA protected osteoblasts from apoptosis induced by oxidative stress. As the activation of caspases occurs, among several factors, by increasing the amount of intracellular ROS, we propose that FucA-GA inhibited apoptosis by decreasing the amount of intracellular ROS (Figure 8). Overall, these data suggest that FucA-GA may have potential therapeutic value for the treatment of disorders of bone formation caused by oxidative stress.

### 2.4. In Vivo Experiments

The in vitro experiments indicated that the conjugation of FucA with GA led to the formation of FucA-GA with better antioxidant activity. Next, we analyzed the antioxidant activity of these compounds in vivo using a zebrafish model.

Zebrafish (*Danio rerio*) is a vertebrate model with a metabolism similar to that of mammals. Therefore, it has been used in various studies, including the evaluation of the antioxidant action of molecules [77]. However, the in vivo studies are preliminary and prompt further studies with zebrafish as well as mammalian models.

**Figure 10:**
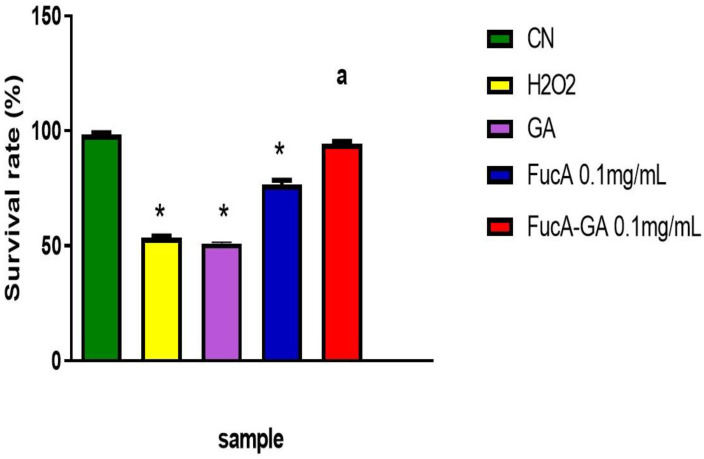
Embryo death analysis after oxidative stress with H_2_O_2_. GA—gallic acid (0.074 mg/mL). NC—negative control composed only of stock water. * indicates a significant difference between NC and samples (* *p* < 0.0001). (a) FucA-GA vs NC (*p* < 0.0001).

The embryo survival rate after exposure to H_2_O_2_ and fucoidans or GA was evaluated. As shown in Figure 10, approximately 50% of the embryos survived after exposure to H_2_O_2_. GA did not affect the toxic activity of the peroxide. We did not find other papers that evaluated the protective action of GA against peroxide in a zebrafish model. Therefore, a comparison with data from other authors was impaired. However, GA (from 1 to 120 µg/mL) has no toxic effect on zebrafish embryos [78]. This rules out the possibility that the concentration used here (0.074 µg/mL) was toxic to the embryos and, therefore, no protective effect was observed for GA.

Embryos exposed to H_2_O_2_ and FucA (0.1 mg/mL) showed significantly higher survival rates (~70%). When treated with FucA-GA (0.1 mg/mL), the survival rate was even higher (~90%). No significant differences were observed compared with the values obtained in the control group. These data indicate that the fucoidans, mainly FucA-GA, conferred protection against the toxic action of H_2_O_2_.

There are some studies on the antioxidant activity of fucoidans in zebrafish [79,80]. The fucoidans evaluated by these studies also promoted the survival of embryos exposed to oxidative stress with fewer dead cells; however, no fucoidan had an effect like that of FucA-GA. 

FucA-GA was superior to FucA in all tests, and it was superior to GA in tests with cells and embryos. Therefore, using a simple, environment-friendly method, we obtained a conjugate (FucA-GA) with antioxidant activity superior to the molecules that gave rise to it (FucA and GA), as well as being superior to those described for other fucoidans.

Regarding the assays with MC3T3 cells, the data indicate the potential of FucA-GA as an adjuvant in the treatment of diseases such as osteoporosis. However, studies with mammals are necessary. Furthermore, studies on the mechanism of the antioxidant action of FucA-GA need to be better clarified. Thus, it would be possible to identify new potential uses for FucA-GA.

## 3. Materials and Methods

### 3.1. Extraction of FucA

The seaweed *Spatoglossum schöederi* was collected at Pirambuzios Beach (5°59′20.6″ S 35°06′50.1″ W), Nísia Floresta-RN, Brazil. The alga was stored in our laboratory, dried at 50 °C under ventilation in an oven, ground in a blender, and incubated with ethanol to eliminate lipids and pigments. The defatted and depigmented seaweeds were then stored in our laboratory and protected from light until polysaccharide extraction. The seaweed was identified based on its morphology [81]. The collection occurred after the authorization of the Brazilian National Management System Genetic Heritage and Associated Traditional Knowledge (loose translation) SISGEN n° A0D4240.

Approximately 100 g of powdered alga was suspended in five volumes (500 mL) of 0.25 M NaCl, and the pH was adjusted to 8.0, using NaOH. Next, 1.5 g of Prolav 750 (Prozyn Biosolutions, São Paulo, Brazil), a mixture of alkaline proteases, was added for proteolytic digestion. After incubation for 18 h at 60 °C, the mixture was filtered through cheesecloth. The resulting extract was referred to as the crude extract and was then subjected to acetone fractionation according to a previously reported method [33]. The extract was filtered, and the filtrate was fractionated by acetone precipitation as follows: ice-cold acetone (0.5 mL) was added to the solution with gentle agitation and maintained at 4 °C for 24 h. The precipitate formed was collected by centrifugation (10,000× *g*, 20 min), dried under vacuum, dissolved in distilled water, and analyzed. The procedure was repeated by adding 0.6, 0.7, 0.9, 1.1, 1.3, and 2.0 volumes of acetone to the supernatant.

The fractions were named according to the volume of acetone used: F0.5v, F0.6v, F0.7v, F0.9v, F1.1v, F1.3v, and F2.0v. 

The FucA purification process was carried out as described in [7]. Briefly, the F0.6v fraction, which contains FucA [7], was subjected to ion exchange chromatography and eluted with increasing concentrations of NaCl. The sample eluted with 1.0 M NaCl, which contained the FucA, was precipitated with methanol (100%, at 4 °C). After 24 h, it was centrifuged, dialyzed, and kept protected from light for future analyses. FucA was used in two studies: this one and in the study of Rodrigues-Souza et al. [82]. Its identity was confirmed by 1HNMR analysis, and the spectra are shown in the previously published paper [82].

### 3.2. Conjugation of GA and FucA

First, 500 mg of FucA was dissolved in acetic acid in water (2% *v*/*v*). Subsequently, 1 mL of 1 M H_2_O_2_ and 0.054 g ascorbic acid were added to this solution. After 30 min, 1.4 mmol of GA was introduced to the reaction and incubated for 24 h. The solution was then centrifuged using an Amicon^®^ Ultra-15 centrifugal filter (Millipore, Burlington, MA, USA) with a 3 kDa cut-off until all unreacted GA was removed [9]. The GA-conjugated FucA solution was named “FucA-GA” and was frozen and lyophilized until future use. 

### 3.3. Dosage of Total Phenolic Compounds, Total Sugar, Protein, and Detrmination of Monossacharide Composition and Molecular Weight

The quantification of phenolic compounds after the covalent binding of GA to FucA was performed using the Folin–Ciocalteau colorimetric method at 765 nm. The result was expressed as %. The control assay was performed using FucA, and the Folin–Ciocalteu procedure was further used to evaluate FucA-GA [32]. The total sugar and proteins contents, monossacharide composition, and molecular weight were determined as described earlier [7].

### 3.4. Assessment of Reducing Power

The reducing power was evaluated after adding 4 mL of the solution containing the samples at different concentrations (0.1, 0.25, 0.5, 1.0, and 2.0 mg/mL) to phosphate buffer (0.2 M; pH 6.6) and potassium ferricyanide (1%). The mixture was incubated for 20 min at 50 °C. The reaction was stopped by the addition of 10% trichloroacetic acid, distilled water, and iron chloride. The absorbance of the solution was measured at a wavelength of 700 nm, and ascorbic acid was used as a control [83].

### 3.5. Evaluation of Hydroxyl (OH) Radical Scavenging

The hydroxyl radicals were generated using 3 mL of sodium phosphate buffer (150 mM, pH 7.4). Phosphate buffer consisted of 10 mM FeSO_4_, 7 H_2_O, 10 mM EDTA, 2 mM sodium salicylate, 30% H_2_O_2_, and different concentrations of FucA and FucA-GA. The radicals were generated as a function of the Fenton reaction (Fe^2+^ + H_2_O_2_ → Fe^3+^ + OH^-^ + OH). The control was phosphate buffer, which replaced hydrogen peroxide. The presence of the hydroxyl radicals was measured at 510 nm after incubation at 37 °C for 1 h. GA was used as the control.

### 3.6. Ferrous Ion-Chelating Ability

The chelating abilities of the samples were evaluated as described earlier [55]. Briefly, each sample at different concentrations was added to the reaction mixture containing FeCl_2_ (0.05 mL, 2 mM) and ferrozine (0.2 mL, 5 mM). The mixture was shaken and incubated for 10 min. at room temperature, and the absorbance of the mixture was measured (562 nm) against a blank (ultrapure water). The control was the reaction mixture without polysaccharides.

The chelating effect was calculated using the corresponding absorbance (A) in the formula given below, where the control is the absorbance in the absence of chelating agents:

Chelating Effect (%) = (^A^control − ^A^sample/^A^control) × 100 (1)

### 3.7. Copper-Chelating Ability

The ability of the extracts to chelate copper ions was determined using the method described by Domazetovic et al. [84]. Pyrocatechol violet, the reagent used in this assay, can associate with certain cations, such as aluminum, copper, bismuth, and thorium. In the presence of chelating agents, this combination is not formed, resulting in decreased staining. This reduction thus allows for the estimation of the chelating activity of copper ions from fucoidans. The test was performed in 96-well microplates with a reaction mixture containing different concentrations of the samples (0.1–2.0 mg/mL), pyrocatechol violet (4 mM), and copper II sulfate pentahydrate (50 mg/mL). The solution in each well was mixed using a micropipette, and the absorbance was measured at 632 nm. The chelating effect was calculated using the corresponding absorbance in the formula given below, where the blank represents the absorbance in the absence of chelating agents.

*Chelating Effect* (%) = (*^A^blank − ^A^sample*/*^A^blank*) × 100 (2)

### 3.8. Determination of Total Antioxidant Capacity

This assay is based on the reduction of Mo(VI) to Mo(V) by sulfated polysaccharides, and the subsequent formation of a green phosphate/Mo(V) complex at an acidic pH [85,86]. Tubes containing sulfated polysaccharides (from 0.001 to 1.0 mg/mL) and reagent solution (0.6 M sulfuric acid, 28 mM sodium phosphate, and 4 mM ammonium molybdate) were incubated at 95 °C for 90 min. After the mixture was cooled to room temperature, the absorbance of each solution was measured at 695 nm against a blank. After performing the experiment, the concentration at which the absorbance values no longer increased was identified. In the case of FucA and FucA-GA, with both polysaccharides, from 0.1 mg/mL no further alterations in the absorbance values were identified. Therefore, this was the concentration chosen to calculate the total antioxidant capacity (TAC), as described earlier [86]. Ascorbic acid was used as standard. 

### 3.9. MTT Assay

For the tests, 5 × 10^3^ cells were grown in 96-well plates with DMEM containing the fucoidans (from 0.1 to 2.0 mg/mL) or GA (from 0.01 to 0.1 mg/mL) for 24 h at 37 °C and 5.0% CO_2_ (each concentration *n* = 6). In the case of GA, the plates were kept in these conditions protected from light. The capacity of the cells to reduce MTT was determined using the colorimetric MTT assay, as described earlier [87].

### 3.10. Induced Oxidative Stress Assay

MC3T3 cells (1 × 10^6^ cells/mL) were seeded in 6-well plates in the presence of 1 mL of Dulbecco’s Modified Eagle Medium (DMEM) supplemented with 10% Fetal Bovine Serum (FCS). After 24 h, the plates were washed, and 1 mL of DMEM supplemented with 10% FCS, sulfated polysaccharides (1.0 mg/mL), and H_2_O_2_ (500 µM, final concentration) were added. The plates were kept under culture conditions (37 °C; 5% CO_2_; dark) for 6 h, and the medium was replaced with 1 mL of the same fresh medium. After 24 h, the cells were subjected to the MTT assay.

### 3.11. Analysis of Cell Proliferation with BrdU Incorporation

The cells (5 × 10^3^ cells/well) were seeded into 96-well plates with 300 μL of fresh medium and incubated for 12 h at 37 °C and 5.0% CO_2_. The medium was removed, the fucoidans (from 0.1 to 2.0 mg/mL) or GA (from 0.01 to 0.1 mg/mL) in DMEM were added, and the plates were incubated for 24 h at 37 °C and 5.0% CO_2_, in the dark. After incubation, the unbound samples were removed by washing the cells twice with 200 μL phosphate buffered saline (PBS), while BrdU incorporation was determined according to the manufacturer’s instructions (BrdU cell proliferation assay kit-Cell Signaling, Danvers, MA, USA). 

### 3.12. Intracellular ROS Production

The levels of intracellular oxygen reactive species were evaluated by quantifying the fluorescence emitted by 2′,7′dichlorofluorescein, the oxidized form of 2′,7′-dichlorofluorescein diacetate (DCFH-DA) (Sigma Chemical Company, St. Louis, MO, USA). MC3T3 cells (3 × 10^5^/well) in 24-well plates) were cultured for 24 h in DMEM with FCS, washed, and then DMEM supplemented with 10% FBS containing H_2_O_2_ (500 µM, final concentration), FucA, and FucA-GA at concentrations of 1.0 mg/mL. The negative control was standardized using DMEM supplemented with 10% FBS. The plates were kept under culture conditions (37 °C; 5% CO_2_; dark) for 6 h. After treatment, the supernatant was discarded, cells were washed with phosphate buffered saline (PBS), and 100 µM DCFH-DA in DMEM containing 1% FBS was added, followed by incubation at 37 °C for 2 h. Then, the DCFH was removed, the cells were washed twice with PBS, and the emitted fluorescence was measured on a flow cytometer (FACS Canto II, BD Biosciences, Eugene, OR, USA) with FACSDiva software, version 6.1.2 (Becton Dickson, Franklin Lakes, NJ, USA). The results were analyzed in FlowJo software (FlowJo, Ashland, OR, USA) and expressed as the ratio of ROS found in cells exposed to samples and ROS found in cells from negative group % (cells exposed only to medium).

### 3.13. Caspase-3 and -9 Activity Assays

MC3T3 cells were cultured for 24 h in DMEM with FCS, washed, and then received medium containing H_2_O_2_ (500 µM, final concentration), FucA, and FucA-GA at concentrations of 1.0 mg/mL. The negative control was standardized using DMEM supplemented with 10% FBS. The cells were cultured for 90 min, then the medium was changed. The analysis pattern was set as 0, 8, 16, and 24 h. Cells were washed in ice-cold PBS with 200 mL of lysis buffer (50 mM Tris-HCl (pH 7.4), 1% Tween 20, 0.25% sodium deoxycholate, 150 mM NaCl, 1 mM EDTA, 1 mM Na_3_VO_4_, 1 mM NaF), and protease inhibitors (1 mg/mL aprotinin, 10 mg/mL leupeptin, and 1 mM 4-(2-aminoethyl)fluoride benzenesulfonyl) for 2 h on ice. Samples were centrifuged, and protein concentrations were determined using Bradford reagent, with bovine serum albumin as the standard. The activities of caspase-3 and -9 proteases in vitro were measured using a caspase activation kit according to the manufacturer’s protocol (Invitrogen, Waltham, MA, EUA). For this, 50 µL of cell lysate was mixed with 50 µL of 2× reaction buffer containing 10 µL of 1 M dithiothreitol and 5 µL of 4 mM synthetic tetrapeptide Asp-Glue-Val-Asp (for caspase 3) or Leu-Glu-His-Asp (for caspase 9) conjugated top-nitroanilide in a 96-well plate, and the mixture was incubated for 2 h at 37 °C in the dark [88].

### 3.14. Zebrafish Embryo Development

We used a wild strain of zebrafish (*Danio rerio*) that were reared domestically. The reproduction was carried out at a ratio of two males to one female, placed in four different breeding tanks. Contact between the animals occurred chemically and visually, with the fish separated by a partition during the night. Mating took place for 1 h in the morning when the divider was removed, ensuring accuracy in the fertilization window and an adequate period for the tests. After fertilization, the eggs were collected and placed in a plastic tray with water for 8 h. The photoperiod of the test was determined to be 24 h (12 h dark/12 h light). All experimental protocols were approved by the Committee for the Use of Animals of the Federal University of Rio Grande do Norte (CEUA 004002/2017).

### 3.15. Embryo Death Analysis after H_2_O_2_-Induced Oxidative Stress

We used the methodology recommended by Kim et al. [89,90] with modifications. The embryos were transferred to a 24-well plate containing 2.0 mL of pond water. Subsequently, they were incubated with GA (0.074 mg/mL), FucA, and FucA-GA (both 0.1 mg/mL), for 1 h, and H_2_O_2_ (500 µM, final concentration) was added to this solution. Embryos were incubated for 24 h at 28 °C, with the H_2_O_2_ solution replaced by stock water. The time and temperature conditions were then repeated. The process was carried out without the interference of light. The protective effects of compounds were evaluated by counting the number of surviving embryos.

### 3.16. Statistical Analyses

All data are expressed as the mean ± standard deviation (*n* = 3) from three observations. Statistical analysis was performed by one-way ANOVA followed by Student’s t-test. Statistical significance was set at *p* < 0.05. Analyses were performed using the GraphPad Prism software version 9.

## 4. Conclusions

In our study, we purified FucA from the seaweed *Spatoglossum schöederi*. The polysaccharide is composed of glucuronic acid, xylose, and fucose, which allowed the conjugation of GA through the redox method to form a compound called FucA-GA. The FucA-GA molecule showed a higher antioxidant activity than FucA, as was evident from the results of TAC and its ability to chelate copper. None of the samples exhibited cytotoxicity on MC3T3 cells. FucA-GA provided the most effective protection to MC3T3 cells against H_2_O_2_-induced stress, through the suppression of ROS production and the induction of apoptosis by ROS. Overall, our data suggested that FucA-GA may have potential therapeutic value for the treatment of bone formation disturbances.

The in vivo and in vitro results suggest that the polysaccharides modified by gallic acid markedly reduce the damage caused by free radicals. Further studies can be conducted to correlate the influence of the structural conformation of FucA with its ability to form phenolic compounds when conjugated with GA.

## Figures and Tables

**Table 1:** Chemical composition of FucA and FucA-GA.

Samples	Sugar%	Phenolic Compounds %	MW kDa	Molar Ratio				
				Fuc	Xil	GlucA	Gal	Sulfate
FucA	77 ± 2	nd	21.0	1	0.32	0.60	nd	1.52
FucA-GA	70 ± 3 *	3.7 ± 0.3 **	20.5	1	0.33	0.55	nd	1.50

Fuc—fucose; Xil—xylose; GlucA—glucuronic acid; Gal—galactose; nd—not detected; FucA—Fucan A obtained from the *S. schröederi*. FucA-GA—Fucan A conjugated with GA. MW—apparent molecular weight. * indicates statistical significance (*p* < 0.05). ** indicates statistical significance (*p* < 0.0001).

## Data Availability

Not applicable.
